# Effects of climate and environment on migratory old people with allergic diseases in China: Protocol for a Sanya cohort study

**DOI:** 10.1016/j.heliyon.2023.e21949

**Published:** 2023-11-10

**Authors:** Qian Hu, Xiufeng Shi, Dan Wang, Yongzhen Huang, Jiashi Gao, Haidao Guan, Han Ren, Xiaoya Lin, Zhaoui Lu, Shilu Tong, Guiyan Yang, Shijian Liu

**Affiliations:** a Department of Hospital Infection, Hainan Branch, Shanghai Children's Medical Center, School of Medicine, Shanghai Jiao Tong University, Sanya, 572022, China; b Department of Science and Education, Hainan Branch, Shanghai Children's Medical Center, School of Medicine, Shanghai Jiao Tong University, Sanya, 572022, China; c Department of Hospital Management, Hainan Branch, Shanghai Children's Medical Center, School of Medicine, Shanghai Jiao Tong University, Sanya, 572022, China; d Big Data Center, Hainan Branch, Shanghai Children's Medical Center, School of Medicine, Shanghai Jiao Tong University, Sanya, 572022, China; e Department of Pediatric Surgery, Hainan Branch, Shanghai Children's Medical Center, School of Medicine, Shanghai Jiao Tong University, Sanya, 572022, China; f Department of Clinical Epidemiology and Biostatistics, Children Health Advocacy Institute, Shanghai Children's Medical Center, School of Medicine, Shanghai Jiao Tong University, Shanghai, 200127, China; g School of Public Health and Social Work, Queensland University of Technology, Brisbane, Australia

**Keywords:** Allergic diseases, Respiratory diseases, Migratory bird-style old people, Environment, Climate

## Abstract

**Background:**

Several studies have reported that the mountain climate can alleviate asthma, however, the effect of tropical climate on migratory elderly, especially in people with respiratory or allergic diseases is unknown.

**Objectives:**

This cohort study aims to explore impact of climate and environmental changes on allergic diseases in migratory old people.

**Methods:**

In this prospective cohort study, we recruited 750 older migratory people, the majority of whom were homeowners to minimize the risk of loss to follow up. The study's inclusion criteria were elderly individuals had moved from northern China to Sanya and suffered from either asthma or allergic diseases. Prior to participation, these individuals provided informed consent and underwent baseline assessment. Subsequently, they will be followed for three years. A face-to-face interview was conducted to gather information regarding their living environment and habits. Trained investigators administered the questionnaires and performed physical examinations including height, weight, and blood pressure, while a professional respiratory doctor conducted pulmonary function tests. Blood samples were promptly tested routine blood test, liver function, kidney function, glucose, triglyceride, allergens, and inflammatory factors. Climate and environmental data were obtained from Sanya Meteorological Bureau and Ecological Environment Bureau, respectively. We primarily compared the differences of participants with asthma or allergic diseases between northern China and Sanya in southern China by Chi-square test, *t*-test or Wilcoxon rank-sum test.

**Findings:**

A total of 750 participants were recruited in this cohort from fourteen communities. All participants were surveyed questionnaires about health and family environment, underwent physical examinations, and collected biological samples for laboratory examinations.

**Novelty:**

This is the first study to evaluate the effects of tropical climate and environment on elderly migrants from cold regions. This study has important implication for the health tourism and aging health, especially for the elderly migrants who suffered the respiratory and allergic diseases. Furthermore, this cohort study establishes a solid foundation for investigating the influence of environmental changes on elderly migrants with allergic diseases.

## Abbreviations

ARIA: Allergic Rhinitis and its Impact on Asthma
AD: Atopic dermatitis
COPD: Chronic obstructive pulmonary disease
FEV_1_: Forced expiratory volume in 1 s
FVC: Forced vital capacity
GOLD: Global Initiative for Chronic Obstructive Lung Disease
MBOP: Migratory bird-style old people
SD: standard deviation
UV: ultraviolet

## 1 Introduction

The latitude China encompasses a wide range, extending from the northernmost Mohe river (53.55° North latitude) to the southernmost Sanya (18.15° North latitude). The northeastern region of China experiences a monsoon and continental climate characterized by cold and dry winters. Conversely, Sanya, located in the southernmost China, exhibits a tropical monsoon climate characterized by warm and humid conditions. For example, the lowest winter temperatures reach approximately −40 °C in the Northeast and 16 °C in Sanya. The impact of these contrasting climates and environments on migratory individuals with respiratory or allergic diseases remains uncertain. Numerous studies conducted across various countries and regions have documented the impact of alterations in temperature and humidity on hospital admissions, life expectancy, and chronic diseases [[1], [2], [3]]. The chronic diseases including respiratory, allergic, endocrine, and cardiovascular diseases, particularly emphasis on respiratory conditions like asthma, chronic obstructive pulmonary disease (COPD), and allergic rhinitis, exhibit heightened sensitivity and substantial susceptibility to environmental conditions [[4], [5], [6]].

Sanya located in the southernmost region of China, possesses a unique tropical island climate. Several studies suggest that lower latitudes are generally associated with higher exposure to ultraviolet (UV) radiation, resulting in and increased incidence of hay fever, dust mites, and mold allergies [7]. Furthermore, UV radiation has been associated with the development of eczema and food allergies [8,9]. Additionally, the tropical and humid environments, indoor dust mites facilitate to breed, and dust mites, which are often the trigger of allergic diseases such as allergic rhinitis and asthma [10,11]. Many studies have confirmed that exposure to PM_10_, PM_2.5_, NO_2_, CO, and O_3_, can deteriorate respiratory diseases [12,13]. Sanya, a famous tourist destination in China, boasts extensive forest coverage, a favorable climate, and excellent air quality, all of which can contribute to the recovery of allergic disorders such as asthma, among the elderly people. Furthermore, the beneficial effects of a tropical climate on migratory individuals with respiratory or allergic diseases who migrate to Sanya poses a new perspective of environment shifts on health.

China has entered a stage of rapid population aging. As of 2019, there were approximately 164.5 million Chinese citizens aged 65 and above, with an additional 26 million citizens aged 80 and above [14]. The city of Sanya, known for its unique tropical climate and excellent air quality, attracts many migratory old people from northern China during the winter-spring season. These individuals, called as migratory bird-style old people (MBOP), generally migrate to Sanya in October or November, and return to their homes in northern China in spring. In 2018, the MBOP in Hainan Province reached 1.3 million, with over one-third of this population originating from the three northeastern provinces of Liaoning, Jilin, and Heilongjiang [15]. The unique climate and high air quality in Sanya are believed to improve the prevalence and severity of chronic diseases such as asthma, and COPD [16,17]. This large-scale and regular population migration is a rare occurrence on a global scale, and the effect of tropical climate on elderly migrants, particularly those with respiratory or allergic disorders remains unknown. However, the following-up of the elderly population over three years in this cohort, which may face great difficulties and challenges. The objective of this cohort study is to explore the impact of unique tropical climate and environmental factors on the MBOP with respiratory or allergic diseases. In addition, this research aims to contribute to the advancement of the health care industry in Sanya, Hainan province.

## 2 Methods

### 2.1 Research philosophy and design

This is a prospective cohort study, with the intention of conducting biannual follow-up over a period of three years. The first follow-up will take place from October to December when MBOP migrate from northern China to Sanya, This timeframe is chosen as it represent a period when temperatures in northern China don't decrease significantly before winter. The second follow-up will occur in March or April before they return to northern China, when temperatures don't obviously increase before summer. Temperature drop increase the risk of respiratory or allergic diseases. We expected to observe and analyze the changes in diseases pattern among participants between the northern region of China and the southern region of Sanya.

### 2.2 Study setting and participants

This study was conducted in Sanya, Hainan Island, a southernmost tourist city with a tropical monsoon climate. The inclusion criteria were as follows: (1) participants who were born and had resided in northern China for a longtime, Northern China encompasses three northeast provinces (Heilongjiang, Jilin, and Liaoning), five north provinces (Beijing, Tianjin, Hebei, Shanxi and Inner Mongolia Autonomous Region), as well as five northwest provinces or autonomous regions (Xinjiang Uygur Autonomous Region, Ningxia Hui Autonomous Region, Qinghai, Gansu, and Shaanxi Provinces); (2) participants were between the ages of 50 and 80 years old; (3) In autumn or winter, participants were willing to migrate to Sanya and subsequently returned to northern China in spring; (4) participants were suffering from COPD, asthma, allergic rhinitis, allergic dermatitis, eczema, urticaria (Table 1). The diagnosis of allergic rhinitis, asthma, eczema, urticarial, or COPD was determined based on self-reported medical history. Allergic rhinitis was defined as a positive response to the question: “Do you have any nasal allergies including hay fever?” according to the guidelines of Allergic Rhinitis and its Impact on Asthma (ARIA) [18]. Asthma was characterized by a self-reported history of asthma, diagnosis by a physician, or the presence of wheeze symptoms within the previous 12 months [19]. Eczema or urticaria was defined on the basis of a self-reported history of asthma, or diagnosis by a physician [20,21]. Atopic dermatitis (AD) was diagnosed by a positive response to the following question: “Have you had an itchy rash at any time in the past 12 months ?” [18] COPD was defined as a post-bronchodilator FEV1/FVC ratia less than 0.7, according to the 2017 guidelines established by the Global Initiative for Chronic Obstructive Lung Disease (GOLD) [22]. Chronic bronchitis was defined as the self-reported production of phlegm for a minimum of three months per year over two consecutive years [23]. The control group was residents who had lived in Sanya for more than three years.

**Table 1:** The groups of migratory bird-style old people (MBOP) in the cohort study.

Number	Groups	Participants	Residence
1	MBOP with asthma	MBOP	Living in Sanya from northern China
2	MBOP with allergic rhinitis		
3	MBOP with Chronic Obstructive Pulmonary Disease (COPD)		
4	MBOP with atopic dermatitis		
5	MBOP with eczema		
6	MBOP with urticaria		

Exclusion criteria included participants who cannot migrate to Sanya for more than one year, and those with communication difficulties or a lack of willingness to cooperate.

### 2.3 Sample size and sampling procedure

The sample size of the cohort study was determined using a one-sided significance level and calculated using an online tool (https://epitools.ausvet.com.au/cohortss), as per a previous study [24], the Forced expiratory volume in 1 s (FEV1) was 92.8 % ± 23.1 and 86.5 ± 26.2 for trial and control groups, respectively. To achieved a type I error α = 0.05, β = 0.10, and account for a loss of follow-up rate of 20 % over a three-year peroid, a cohort of 750 participants would be required. The 750 MBOP participants were recruited from fourteen communities where the MBOP living in concentrated. We recruited the participants who have minium risk of loss to follow-up for majority of them were homeowners and moved to Sanya every year. The selection of these fourteen communities was based on their feasibility for recruiting suitable participants. Due to the lack of information regarding the distribution of communities where MBOP live in the entire city, random sampling is not a applicable approach.

### 2.4 Data sources and collection methods

#### 2.4.1 Ethics approval

The study was conducted in accordance with the Declaration of Helsinki, and the protocol was approved by the Institute Review Board of Sanya Women and Children Hospital (approval number: SYFYIRB2022009). All participants signed informed consent and willingly agreed to participate in the study. Furthermore, this study has been registered on the Clinical Trials website (ClinicalTrials.gov) under the iIdentifier NCT05330637.

#### 2.4.2 Investigation process

**Fig. 1:**
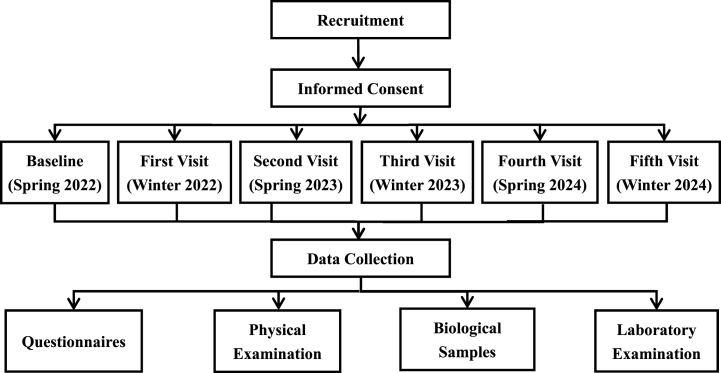
Flowchart of the prospective cohort study.

This study started on March 1, 2022, and the baseline survey was completed on March 31, 2022. The follow-up in 2023 is currently ongoing. In order to gather participants, a recruitment notice was disseminated within various communities, inviting eligible individuals to take part in this study. Prior to their involvement, volunteers were informed of the comprehensive information regarding the objectives, content, as well as their rights and responsibilities within the study. After they signed the informed consent, participants underwent a series of sequential procedures, including face-to-face interview using questionnaires, physical examinations, blood sample collection, and laboratory analyses. The flowchart illustrating the progression of this study is depicted in Fig. 1.

### 2.5 Data collection

The survey included questionnaire, physical examinations, biological samples collection, and laboratory examinations. Table 2 shows the contents required in this study.

**Table 2:** The content required at baseline, follow-up of the prospective cohort study.

Items	Baseline and first visit（2022）	Second and third visit（2023）	Fourth and fifth visit（2024）
Health and family environment questionnaire (all participants)			
Basic information	✓		
Medical history	✓		
Symptoms of allergic diseases	✓	✓	✓
Behavioral Habits	✓		
Environmental Exposures	✓	✓	✓
**Physical examination**			
Height (all participants)	✓	✓	✓
Weight (all participants)	✓	✓	✓
Blood pressure (all participants)	✓	✓	✓
Lung function (Patients with asthma or COPD)	✓	✓	✓
**Biological samples (all participants)**			
Veinal blood	✓	✓	✓
**Laboratory examination**			
Routine blood test	✓	✓	✓
Liver function	✓	✓	✓
Renal function	✓	✓	✓
Glucose	✓	✓	✓
Triglyceride	✓	✓	✓
Allergens	✓	✓	✓
Inflammatory factors	✓	✓	✓
**Climatic and environmental data**			
Temperature	✓	✓	✓
Humidity	✓	✓	✓
Wind speed	✓	✓	✓
Sunshine	✓	✓	✓
SO_2_	✓	✓	✓
CO_2_	✓	✓	✓
NO_2_	✓	✓	✓
O_3_	✓	✓	✓
CO	✓	✓	✓
PM_10_	✓	✓	✓
PM_2.5_	✓	✓	✓

COPD: Chronic Obstructive Pulmonary Disease.

#### 2.5.1 Questionnaire investigation

Trained investigators performed face-to-face interview to administer questionnaires aimed at collecting personal information, residence environment details, migration behavior, and health status. Personal information mainly included age, gender, education, occupation, and so on. The living environment mainly focused on housing conditions, floor, proximity to main roads or industrial zones, exposure to dust, indoor plants or pets, drinking water sources, daily fuel usage, ventilation, humidity, and cleaning habits. The study focused primarily on the health status at different migrating timeline and location. The health status assessment mainly considered factors such as family history, symptoms, frequency of allergic diseases, and changes of diseases after relocating to Sanya.

#### 2.5.2 Physical examination

Physical examination included measurements of height, weight, blood pressure and pulmonary function (FEV1 and FVC) as outlined in Table 2. These physical examinations were carried out during the initial assessment and subsequent follow-up visits. Height and weight were measured by the investigator while participants were dressed in light clothing and barefoot. Blood pressure was measured using an electronic sphygmomanometer. Pulmonary function was performed by the respiratory physician in accordance with established standard operating procedures. A pulmonary function FEV1/FVC ratio below 70 % is indicative to COPD.

#### 2.5.3 Biological samples and laboratory examination

Nurses collected three tubes of venous blood samples (5–10 mL) from participants before their breakfast. The collected blood samples were promptly transported to the local clinical laboratory for processing within 2 h. Subsequently, the biological samples were centrifuged and stored in a refrigerator at - 80 °C. The whole blood sample was used for a routine blood test and serum was tested and analyzed for liver function, kidney function, glucose levels, triglyceride, allergens, and inflammatory factors (as detailed in Table 2).

#### 2.5.4 Climatic and environmental data

Climate and environmental data were obtained from the Sanya Meteorological Bureau and Sanya Ecological Environment Bureau, respectively. Meteorological data primarily included temperature, humidity, wind speed, and sunshine, while air pollution parameters encompassed SO_2_, CO_2_, NO_2_, O_3_, CO, PM_10_, and PM_2.5_.

### 2.6 Data analysis

Table 3 presents the primary and secondary outcomes observed in the cohort study. The primary outcome focuses on the difference of MBOP with asthma, COPD, allergic rhinitis or atopic dermatitis between the winter-spring season in tropical Sanya, Southern China, and the summer-autumn season in northern China. The secondary outcomes include various factors such as asthma control, asthma-related life quality, lung function, and allergens. Categorical variables are expressed as frequencies and were analyzed by chi-square testing to compare intergroup differences. The study will be used *t*-test, analysis of variance, and Wilcoxon rank-sum test to assess disparities in the distribution of continuous variables. These variables will be characterized by mean ± standard deviation (SD) for normally distributed data or median with interquartile range for non-normally distributed data.

**Table 3:** The outcomes in the cohort study.

Number	Category	Outcomes	Description
1	Primary Outcome	The difference of MBOP with asthma, COPD, allergic rhinitis or atopic dermatitis between winter-spring season in tropical Sanya, Southern China and summer-autumn season in northern China.	The asthma, COPD, allergic rhinitis or atopic dermatitis will be diagnosed and assessed through history of diseases, questionnaire, and lung function test.
2	Secondary	Asthma control of MBOP with asthma.	Quality of life is assessed through Asthma Control Questionnaire (ACQ).
3	Outcome	Asthma life quality of MBOP with asthma.	Quality of life is assessed through Asthma Quality of Life Questionnaire (AQLQ).
4		Lung function of MBOP with asthma or COPD.	Lung function is assessed through simple lung function test using spirometer.
5		The respiratory function of MBOP with COPD.	Lung function is assessed through Saint George Respiratory Questionnaire (SGRQ).
6		Allergens were detected in MBOP with asthma, allergic rhinitis or atopic dermatitis.	Veinal blood was extracted to detect allergens.
7		Inflammatory factors will be detected in MBOP with asthma, allergic rhinitis or atopic dermatitis.	Inflammatory factors such as esophageal granulocytes and eosinophils will be detected to assess inflammatory status of the MBOP.
8		The change of asthma incidence rate in MBOP.	Diagnosis and severity grading of asthma is based on the “American Thoracic Society” or the “National Heart, Lung, and Blood Institute guidelines.”

COPD: Chronic Obstructive Pulmonary Disease.

## 3 Discussion

This prospective cohort study investigates the impact of environmental changes on respiratory or allergic diseases among elderly migrants in Sanya, and is expected to provide evidence for the future advancement of Sanya's tourism and health care industry. Previous epidemiological studies mainly focused on the impact of climate and environment on the health of local populations, specifically pertaining to conditions such as asthma, eczema, and COPD [13,25,26]. This study innovatively explores the impact of tropical climate on respiratory and allergic diseases among a large-scale population of migrants who have relocated from northern China to Sanya in southern China over long distances.

Notably, there exists a significant contrast in climatic and environmental circumstance between the northern and southern regions of China. Numerous studies have found that climatic and environmental factors affect physical health [27,28]. Especially in winter, temperature of the southernmost region of Sanya is much higher than that in northern China. Yixiang Zhu et al. reported sudden temperature drop significantly increases the risk of asthma exacerbation in cold weather [29]. Consequently, MBOP migrating from northern China to Sanya may experience slight temperature drop, potentially mitigating the risk of respiratory and allergic diseases such as asthma. Additionally, there are notable distinctions in air quality, sunshine, and ultraviolet radiation levels between the two regions, with Sanya exhibiting superior air quality compared to northern China [30]. Previous studies have indicated that the mountain environment can alleviate asthma symptoms, possibly due to reduced pollution levels and less allergens [24,31].

The primary objective of this study is to evaluate the influence of the tropical climate and environment on allergic diseases among elderly individuals who have migrated to Sanya. This assessment will monitor changes in symptoms, serological indicators, and lung function. By conducting through investigations, we will able to determine the prevalence of diseases and observe alterations in symptoms within the MBOP population, as well as obtain serological indicators such as allergens, and analyze the risk factors from their living environment, and exposure sources. The study aims to provide scientific evidence regarding the potential alleviation of allergic and respiratory diseases among the MBOP population. Given the growing aging population in China and a large number of MBOP gathering in Sanya, this research can additionally serve as a foundation for the government to enhance the provision of health management services for the MBOP community.

Globally, there are a scarcity of studies on the impact of tropical climates on respiratory and allergic diseases [[32], [33], [34]], particularly in relation to elderly population who regularly migrate and reside in a new environments for a relatively longtime. Consequently, this study serves as a valuable foundation for the planning and implementation of healthcare services for migrating populations in southern China. In addition, it offers pertinent insights for cities in southern China seeking to attract and cultivate tourism, and health care industries. However, we acknowledge the limitations in this study. The MBOP were recruited from the local community, with most of them being homeowners. Notably, their high socioeconomic status may limit the generalizability of the findings to general MBOP population. Second, MBOP were recruited from various regions in northern China. This study cannot objectively assess diseases status of all MBOP in local medical institutions of northern China. Furthermore, it is worth mentioning that there was no control group in this study, and instead, a self-comparison method was utilized to evaluate changes before and after their arrival in Sanya.

## 4 Conclusions and implication

The study holds significant implications for the fields of health tourism and aging health, especially for migratory elderly population who suffer respiratory and allergic diseases. It has potential to contribute to the development of a new theoretical framework surrounding climate therapy, specifically examining the impact of tropical climates on migratory residents with respiratory or allergic diseases originating from colder regions. Furthermore, the findings may have broader benefits for other diseases, including cardiovascular and endocrine disorders.

## Study status

Prerecruitment and baseline survey data collection for older migrants was completed in March 2022. First follow-up visit had been finished in December 2022.

## Ethics statement

The study was conducted in accordance with the Declaration of Helsinki, and the protocol was approved by the Ethics Committee of Sanya Women and Children Hospital (number: SYFYIRB2022009). All participants signed informed consent and agreed to participate.

## Funding

This work was supported by the Project of “Unveiling the Top” [grant number SYFY-JBGS-202201], 10.13039/501100001809National Science Foundation of China [82173534, 81872637].

## Data availability statement

Data will be made available on reasonable request.

## CRediT authorship contribution statement

**Qian Hu:** Writing – original draft. **Xiufeng Shi:** Data curation, Investigation, Methodology, Project administration, Supervision. **Dan Wang:** Data curation, Investigation, Project administration, Resources, Supervision. **Yongzhen Huang:** Investigation, Project administration. **Jiashi Gao:** Investigation. **Haidao Guan:** Investigation. **Han Ren:** Investigation. **Xiaoya Lin:** Data curation, Investigation, Zhaohui Lu, Conceptualization, Project administration, Supervision. **Shilu Tong:** Conceptualization, Supervision, Writing – review & editing. **Guiyan Yang:** Conceptualization, Investigation, Project administration, Supervision, Writing – review & editing. **Shijian Liu:** Conceptualization, Data curation, Formal analysis, Funding acquisition, Investigation, Methodology, Project administration, Resources, Supervision, Validation, Writing – original draft, Writing – review & editing.

## Declaration of Competing Interest

The funding sponsors had no role in the design of the study; in the collection, analyses, or interpretation of data; in the writing of the manuscript, and in the decision to publish the results.

## References

[1] Bunker A., Wildenhain J., Vandenbergh A., Henschke N., Rocklov J., Hajat S., Sauerborn R. (2016). Effects of air temperature on climate-sensitive mortality and morbidity outcomes in the elderly; a systematic review and meta-analysis of epidemiological evidence. EBioMedicine.

[2] Dastoorpoor M., Khodadadi N., Masoumi K., Khanjani N., Idani E., Borsi S.H., Goudarzi G., Raji H., Sharafkhani R. (2021). Physiological equivalent temperature (PET) and non-accidental, cardiovascular and respiratory disease mortality in Ahvaz, Iran. Environ. Geochem. Health.

[3] Gronlund C.J., Zanobetti A., Wellenius G.A., Schwartz J.D., O'Neill M.S. (2016). Vulnerability to renal, heat and respiratory hospitalizations during extreme heat among U.S. Elderly. Clim. Change.

[4] Correia Junior M.A., Sarinho E.S., Rizzo J.A., Sarinho S.W. (2017). Lower prevalence and greater severity of asthma in hot and dry climate. J. Pediatr..

[5] Pesce G., Bugiani M., Marcon A., Marchetti P., Carosso A., Accordini S., Antonicelli L., Cogliani E., Pirina P., Pocetta G. (2016). Geo-climatic heterogeneity in self-reported asthma, allergic rhinitis and chronic bronchitis in Italy. Sci. Total Environ..

[6] Wang Z., Zhou Y., Luo M., Yang H., Xiao S., Huang X., Ou Y., Zhang Y., Duan X., Hu W. (2020). Association of diurnal temperature range with daily hospitalization for exacerbation of chronic respiratory diseases in 21 cities, China. Respir. Res..

[7] Oktaria V., Dharmage S.C., Burgess J.A., Simpson J.A., Morrison S., Giles G.G., Abramson M.J., Walters E.H., Matheson M.C. (2013). Association between latitude and allergic diseases: a longitudinal study from childhood to middle-age. Ann. Allergy Asthma Immunol..

[8] Osborne N.J., Ukoumunne O.C., Wake M., Allen K.J. (2012). Prevalence of eczema and food allergy is associated with latitude in Australia. J. Allergy Clin. Immunol..

[9] Suaini N.H., Zhang Y., Vuillermin P.J., Allen K.J., Harrison L.C. (2015). Immune modulation by vitamin d and its relevance to food allergy. Nutrients.

[10] Carrard A., Pichler C. (2012). House dust mite allergy. Ther. Umsch..

[11] Wang J., Zhang Y., Li B., Zhao Z., Huang C., Zhang X., Deng Q., Lu C., Qian H., Yang X. (2021). Asthma and allergic rhinitis among young parents in China in relation to outdoor air pollution, climate and home environment. Sci. Total Environ..

[12] Duan R., Niu H., Yu T., Huang K., Cui H., Chen C., Yang T., Wang C. (2021). Adverse effects of short-term personal exposure to fine particulate matter on the lung function of patients with chronic obstructive pulmonary disease and asthma: a longitudinal panel study in Beijing, China. Environ. Sci. Pollut. Res. Int..

[13] Wang J., Zhang Y., Li B., Zhao Z., Huang C., Zhang X., Deng Q., Lu C., Qian H., Yang X. (2022). Eczema, facial erythema, and seborrheic dermatitis symptoms among young adults in China in relation to ambient air pollution, climate, and home environment. Indoor Air.

[14] Fang E.F., Xie C., Schenkel J.A., Wu C., Long Q., Cui H., Aman Y., Frank J., Liao J., Zou H. (2020). A research agenda for ageing in China in the 21st century (2nd edition): focusing on basic and translational research, long-term care, policy and social networks. Ageing Res. Rev..

[15] Xing Q. (2019).

[16] Yan P., Liu P., Lin R., Xiao K., Xie S., Wang K., Zhang Y., He X., Zhao S., Zhang X. (2019). Effect of ambient air quality on exacerbation of COPD in patients and its potential mechanism. Int. J. Chronic Obstr. Pulm. Dis..

[17] Wang Y. (2017).

[18] Wang X.D., Zheng M., Lou H.F., Wang C.S., Zhang Y., Bo M.Y., Ge S.Q., Zhang N., Zhang L., Bachert C. (2016). An increased prevalence of self-reported allergic rhinitis in major Chinese cities from 2005 to 2011. Allergy.

[19] Huang K., Yang T., Xu J., Yang L., Zhao J., Zhang X., Bai C., Kang J., Ran P., Shen H. (2019). Prevalence, risk factors, and management of asthma in China: a national cross-sectional study. Lancet.

[20] Leshem Y.A., Chalmers J.R., Apfelbacher C., Katoh N., Gerbens L.A.A., Schmitt J., Spuls P.I., Thomas K.S., Howells L., Williams H.C. (2022). Measuring atopic eczema control and itch intensity in clinical practice: a consensus statement from the harmonising outcome measures for eczema in clinical practice (home-cp) initiative. JAMA Dermatol..

[21] Ellwood P., Asher M.I., Bjorksten B., Burr M., Pearce N., Robertson C.F. (2001). Diet and asthma, allergic rhinoconjunctivitis and atopic eczema symptom prevalence: an ecological analysis of the International Study of Asthma and Allergies in Childhood (ISAAC) data. ISAAC Phase One Study Group. Eur. Respir. J..

[22] Vogelmeier C.F., Criner G.J., Martinez F.J., Anzueto A., Barnes P.J., Bourbeau J., Celli B.R., Chen R., Decramer M., Fabbri L.M. (2017). Global strategy for the diagnosis, management, and prevention of chronic obstructive lung disease 2017 report. GOLD Executive Summary. Am. J. Respir. Crit. Care Med..

[23] Miele C.H., Jaganath D., Miranda J.J., Bernabe-Ortiz A., Gilman R.H., Johnson C.M., Diette G.B., Wise R.A., Checkley W. (2016). Urbanization and daily exposure to biomass fuel smoke both contribute to chronic bronchitis risk in a population with low prevalence of daily tobacco smoking. COPD.

[24] Rijssenbeek-Nouwens L.H., Fieten K.B., Bron A.O., Hashimoto S., Bel E.H., Weersink E.J. (2012). High-altitude treatment in atopic and nonatopic patients with severe asthma. Eur. Respir. J..

[25] Zhou J., Lei R., Xu J., Peng L., Ye X., Yang D., Yang S., Yin Y., Zhang R. (2022). The effects of short-term pm2.5 exposure on pulmonary function among children with asthma-A panel study in Shanghai, China. Int. J. Environ. Res. Public Health.

[26] Bourbeau J., Doiron D., Biswas S., Smith B.M., Benedetti A., Brook J.R., Aaron S.D., Chapman K.R., Hernandez P., Maltais F. (2022). Ambient air pollution and dysanapsis: associations with lung function and chronic obstructive pulmonary disease in the canadian cohort obstructive lung disease study. Am. J. Respir. Crit. Care Med..

[27] Ou C.Q., Song Y.F., Yang J., Chau P.Y., Yang L., Chen P.Y., Wong C.M. (2013). Excess winter mortality and cold temperatures in a subtropical city, Guangzhou, China. PLoS One.

[28] D'Amato G., Vitale C., De Martino A., Viegi G., Lanza M., Molino A., Sanduzzi A., Vatrella A., Annesi-Maesano I., D'Amato M. (2015). Effects on asthma and respiratory allergy of Climate change and air pollution. Multidiscip. Respir. Med..

[29] Zhu Y., Yang T., Huang S., Li H., Lei J., Xue X., Gao Y., Jiang Y. (2022). Cold temperature and sudden temperature drop as novel risk factors of asthma exacerbation: a longitudinal study in 18 Chinese cities. Sci. Total Environ..

[30] Yan P., Liu P., Lin R., Xiao K., Xie S., Wang K. (2019). Effect of ambient air quality on exacerbation of COPD in patients and its potential mechanism. Int. J. Chron. Obstruct. Pulmon. Dis.

[31] Fieten K.B., Rijssenbeek-Nouwens L.H., Hashimoto S., Bel E.H., Weersink E.J. (2019). Less exacerbations and sustained asthma control 12 months after high altitude climate treatment for severe asthma. Allergy.

[32] Larenas-Linnemann D., Michels A., Dinger H., Shah-Hosseini K., Mosges R., Arias-Cruz A., Ambriz-Moreno M., Barajas M.B., Javier R.C., de la Luz Cid Del Prado M. (2014). Allergen sensitization linked to climate and age, not to intermittent-persistent rhinitis in a cross-sectional cohort study in the (sub)tropics. Clin. Transl. Allergy.

[33] Marinko Š., Domingo R.S., Małgorzata P.R. (2021). Impact of COVID-19 on the travel and tourism industry. Technol. Forecast. Soc. Change.

[34] Walstra K.M., Widmer M., Busato A. (2006). Seasonal variation in orthopedic health services utilization in Switzerland: the impact of winter sport tourism. BMC Health Serv. Res..

