# Supplementary material for: Effects of climate and environment on migratory old people with allergic diseases in China: Protocol for a Sanya cohort study
Source: Heliyon. 2023 Nov 10;9(11):e21949. doi: 10.1016/j.heliyon.2023.e21949 (PMC10692782; doi:10.1016/j.heliyon.2023.e21949)
Supplement: Multimedia component 1 [file mmc1.pdf]

Investigator: ①HR( ) ②QH( ) ③HDG( ) ④DYC( ) ⑤YCH( ) ⑥KQZ( ) ⑦yourself( ) ⑤other:

Middle Aged and Elderly Health Questionnaire

Dear middle aged and elderly friends, Register No.______ Name: _________, Gender: ① Male ② Female, Date of birth: _____Year___Month___Day

1. Original residence: ①Heilongjiang Province ②Jilin Province ③Liaoning Province ④Inner Mongolia ⑤Xinjiang ⑥Gansu Province ⑦Shaanxi Province ⑧Beijing ⑨Hebei Province ⑩Tianjin Province (11)Shanxi Province (12)Other: Province；(13) Reside for more than 5 years in local Sanya

2. Are you currently suffer from the following diseases (query the health situation in accordance with the history of the disease)

①Asthma( ) ②Chronic Obstructive Pulmonary Disease (COPD) ( ) ③Allergic rhinitis /Pharyngitis( ) ④Eczema /Urticaria /Atopic dermatitis( ) ⑤ Food / Drug Allergy( ) ⑥ Bronchitis / Bronchiectasis( ) ⑦ Pulmonary Emphysema ( ) ⑧Hypertension( ) ⑨ Diabetes Mellitus( ) ⑩Other ____________

3. List of physical examination, please tick or fill in the results after the completed item.

A. Height______cm, B. Weight:_____kg ; C. Blood pressure: systolic pressure mmHg, diastolic pressure: mmHg; D. Lung fuction: ( )

Section 1 Basic information

4. Current residence: Jiyang District, Sanya ①Zuoan Fenghuang Shuicheng ②Cuiping Fenghuang Shuicheng ③Wanke Hupan ④Yishanhu ⑤Youlanhu ⑥Ziyuetai ⑦Others__。

5. You have lived in Hainan Province over 3 months for___times, in which Sanya for____times, Normally move in at month, and move out at____month next year.

6. The apartment you living in was bought or rent: ①bought: by yourself or son and daughters( ), other relationships( ), ②rent

7. Your degree of education: ①Illiterate or semi-literate ②Elementary school ③Junior high school ④Secondary school, technical school ⑤High school ⑥Junior college ⑦Undergraduate ⑧Postgraduate (master or doctorate)

8. Your occupation before retirement: ①Organ administrative personnel; ②Enterprise managers; ③Private business owners; ④Professional and technical personnel; ⑤Ordinary staff; ⑥Self-employed; ⑦Commercial service personnel; ⑨ Farmers, forestry, fishing, animal husbandry, water conservancy production personnel; ⑩ Unemployed

9. Will you always check Wechat group information or message: ①Yes ②No; How you would like to be notified of the next survey: ①Wechat group information ( ) ②phone call

Your contact number (very important):___________; Your family member contact number(very important):___________

Section 2 Past medical history and symptoms of allergic diseases

Personal and family past medical history

10. Do you have respiratory diseases or chronic cough before the age of 18? ( ), if you do, ①before 12-year-old( ) ②after 12-year-old ( )

11. Do your family members have a history of the following respiratory diseases: ①Asthma( ) ②Chronic Obstructive Pulmonary Disease (COPD) ( ) ③Allergic rhinitis( ) ④Eczema / Atopic dermatitis( ) ⑤ Food / drug allergy ⑥

Bronchitis / Bronchiectasis( ) ⑦ Pulmonary Emphysema ( ) ⑧ Other respiratory diseases.

Existing disease status (please only fill in the corresponding disease in the medical history)

Ⅰ Your health condition about asthma

12. Do you have any of the following symptoms while living in the North: ①Wheezing or whistling in the chest; ② Waking up at night due to chest tightness, shortness of breath, or a coughing attack

13. How has your asthma condition changed since you arrived in Sanya compared with the north:

① Significantly relieved ② Somewhat relieved ③ No significant change ④ Aggravated ⑤ Significantly worsened

If you choose “significantly relieved” or “somewhat relieved”, it means the symptoms are relieved: ( ), times of attacks is reduced: ( ), the medication is reduced or stopped: ( )

Ⅱ Your health condition about Chronic Obstructive Pulmonary Disease (COPD)

14. Do you have any of the following respiratory symptoms: ① Cough ( ) ② Expectoration ( ) ③ Wheezing ( ) ④ Dyspnea ( ) ⑤ Hemoptysis ( ) ⑥ Other ( )

15. How has your Chronic Obstructive Pulmonary Disease (COPD) condition changed since you arrived in Sanya compared with the north:

① Significantly relieved ② Somewhat relieved ③ No significant change ④ Aggravated ⑤ Significantly worsened

If you chose “significantly relieved” or “somewhat relieved”, it means: the symptoms are relieved: ( ), times of attacks is reduced: ( ), the medication is reduced or stopped: ( )

Ⅲ Your health condition about allergic rhinitis /pharyngitis

16. When living in the original residence in the north, in the absence of a cold or flu, have you ever experienced: ① choking ( ) ② runny nose ( ) ③ nasal congestion ( ) ④ itchy nose ( ) ⑤ itchy eyes ( ) ⑥ dry eyes ( ) ⑦ cough ( ) ⑧ expectoration ( ) ⑨ dry throat ( ) ⑩ sore throat ( )

17. Do those symptoms : ①interfere with sleep? ( ) ②limit daily activities (sports, leisure)? ( ) ③bother you? ( )

18. Your nasal or throat symptoms occur ____days a week on average, or how the symptoms were at the worst?

19. When living in the original residence in the north, how long could your nasal or throat symptoms last: ① <4 weeks ② >4weeks

20. Generally speaking, have you had nasal or pharyngeal symptoms for_____years?

21. Do you have nasal symptoms at the same season every year? ( ) If yes, the symptoms worsen at_month every year?

22. Do you know what will cause your nose or throat symptoms? (Multiple choices are allowed)

① exposed to plants or pollen ② exposed to cold air ③ contact with pets ④ exposed to paint, dust and smoke ⑤ others

23. When living in the original residence in the north, have you ever been to a hospital or clinic because of allergic rhinitis or pharyngitis? ( )

24. You have received: ① surgical treatment ② drug treatment ③ none because of allergic rhinitis or pharyngitis. How do you feel after treatment? ① Effective ② ineffective ③ no significant effect

25. Are you still on medication? ①Yes ②No

26. Are your nose or throat problems with any of the following conditions? ① Asthma ② Eczema ③ Nasal

polyps/sinusitis ④ Allergic cough

27. Are your family bother by allergic disease? ①Yes ②No

28.How has your allergic rhinitis or pharyngitis condition changed since you arrived in Sanya compared with the north:

① Significantly relieved ② Somewhat relieved ③ No significant change ④ Aggravated ⑤ Significantly worsened

If you chose “significantly relieved” or “somewhat relieved”, it means : the symptoms are relieved: ( ), times of attacks is reduced: ( ), the medication is reduced or stopped: ( )

29. Do you often swim: ① Yes ② No; Does rhinitis worsen after swimming: ① Yes ② No

Ⅳ **Your health condition about Eczema /Urticaria /Atopic dermatitis**

30. Have you ever had or been diagnosed with eczema? ①Yes ②No

31. Have you ever had or been diagnosed with urticaria? ①Yes ②No

32. Have you been diagnosed with other types of dermatitis besides eczema and urticaria? ①Yes ②No

33. Have you ever had repeated skin itching or joint flexion rash in the past ①Yes ②No; Are there rash on other parts: ①Yes ②No

a. If yes, this symptom first occurred when you were_____years old

b. If yes, is this symptom related to food, environment changing, weather changing, etc. ①Yes ②No

c. In the last 12 months, have you ever had repeated skin itching or rash on the flexor side of the joint ①Yes ②No

34. How has your eczema /urticaria /atopic dermatitis condition changed since you arrived in Sanya compared with the north:

① Significantly relieved ② Somewhat relieved ③ No significant change ④ Aggravated ⑤ Significantly worsened

If you chose “significantly relieved” or “somewhat relieved”, it means : the symptoms are relieved: ( ), times of attacks is reduced: ( ), the medication is reduced or stopped: ( )

Ⅴ **Your health condition about food / drug allergy**

35. When living in the original residence in the north, have you ever been diagnosed with a food allergy by a doctor ① Yes ②No

36. Have you ever been tested for allergens? ①Yes ②No

37. Have the following food allergies occurred in the allergen testing or in your life? ①Milk or dairy products ②Eggs ③Fish, shrimps and crabs ④Nuts ⑤Wheat ⑥Beef ⑦Beans or soy products ⑧Fruits ⑨Vegetables ⑩Others

38. Have you ever received antibiotics such as penicillin? (Multiple choices)

（1）① No ② Yes,（2）if yes, it occurred ①under 1 year old ②1-2 years old ③ After 3 years;（3）__ treatments like this?

39. Have you ever been diagnosed with a drug allergy by your doctor? ①Yes ② No

40. Have you had any symptoms of drug allergy ①Yes ② No

If you choose “Yes”, it was (Multiple choices): a. Penicillin ( ) b. Cephalosporins ( ) c. Sulfonamides ( ) d. Others ( ) e. Uncertain ( ) that caused those symptoms.

Ⅵ **Your health condition about bronchitis / bronchiectasis/ pulmonary emphysema**

41. When living in the original residence in the north, have you had a cough or sputum for more than 3 months in each of the 2 consecutive years? ①Yes ( ) ②No ( )

42. Have you ever been diagnosed with bronchiectasis or pulmonary emphysema in a hospital or doctor? ①Yes ( ) ② No ( )

43. How has your bronchitis / bronchiectasis/ pulmonary emphysema condition changed since you arrived in Sanya compared with the north :

① Significantly relieved ② Somewhat relieved ③ No significant change ④ Aggravated ⑤ Significantly worsened

If you chose “significantly relieved” or “somewhat relieved”, it means: the symptoms are relieved: ( ), times of attacks is reduced: ( ), the medication is reduced or stopped: ( )

Ⅶ Your health condition about hypertension

44. How has your hypertension condition changed since you arrived in Sanya compared with the north:

① Blood pressure returned to normal ② blood pressure decreased but still high ③ no significant change ④ blood pressure increased

Whether to take antihypertensive drugs: ① stop taking drugs after blood pressure drops ( ) ② reduce the dosage of blood pressure drops ( ) ③ the dosage does not change ( )

Section 3 Behavioral Habits and Environmental Exposures

Ⅰ Behavioral Habits

45. How often do you eat seafood every week: ① not eating ② less than 1 time / month ③ less than 1 time / week ④ 1-3 times / week ⑤ 4-6 times / week ⑥ 1 time / day

46. Do you smoke? ① Used to smoked for about____years, smoke____cigarettes a day in the past, but quit smoking now ② Currently smoking, have been smoking for____years, smoke____cigarettes a day; ③ No smoking or passive smoking; ④ No smoking, but someone in the family or workplace often smokes (passive smoking).

47. Do you exercise every day? ① Walking____×10,000 steps/day or____hours; ②Dancing ( ) ③ Tai Chi ( ) ④ Others ( )

48. You go to bed at___p.m. every night, get up at___a.m. in the morning, and take a nap for___hours every day.

Ⅱ The environment of original residence and work place in the north

49. Have you ever been exposed to occupational dust or gas for a long time in your previous work ①Yes, ②No

50. Traffic conditions within 50 meters around the house in the original place of residence ①Close to the main traffic road ②Community far from the traffic trunk ③Remote residential area

51. Type of housing in the original place of residence: ①Building, in___floor. ②Building by yourself ③Bungalow ④ Villa ⑤Others

52. What is the main source of drinking water in your home? ① Tap water ② Mountain spring water ③ Bottled pure water ④ Water treated by water purifier ⑤ Well water ⑥ Groundwater

53. Do you have flowers and plants indoors in your original residence? ①Yes ②No; 50. Do you have plush toys in your home ①Yes ②No

54. What fuel does your kitchen or heating use? ①Central heating, etc.; ②Air conditioning or floor heating; ③Modern fuels, such as liquefied gas, gas, natural gas; ④ Biofuels, such as wood, crops, animal manure or charcoal, etc.;

55. The ventilation in your kitchen ①very poor ②average ③good ④very good

56. In the past year, has there been a musty or pungent smell in your home? ①No ②Almost every day ③Occasionally ④Only in rainy days ⑤Only in the rainy season

57. Do you currently have pets in your home? ①No ②Yes; Have you disposed of pets due to allergic diseases of family members? ①Yes ②No

58. Have you ever seen mice or cockroaches in your home in the past year? ①Never ②Rarely ③Few ④A lot

59. How often do you usually clean the room (including vacuuming, sweeping, mopping, etc.)?

①Daily ②4-6 times a week ③1-3 times a week ④1-3 times a month ⑤Less than once a month

60. How often do you wash the curtains in your home? ①Never cleaned ②Monthly ③Quarterly ④Six months ⑤ One year or more

Informed Consent

Dear resident friends:

We invite you to participate in a project called " Study on the Influence of Climatic and Environmental Factors on Respiratory Diseases in Sanya". Before you decide whether to participate in this research, please read the following carefully, it can help you understand the research purpose, research content, research protocol and duration of the research, as well as the possible benefits of participating in this research. benefit, risk or discomfort. You can discuss with your relatives, friends, or ask the project team staff for more detailed instructions to help you decide whether to join the study.

1. What is the purpose of this research?

Asthma and allergic diseases are threatening human health. This project analyzes the impact of climate and environmental factors in Sanya on middle-aged and elderly asthma and allergic diseases; compares the impact of different climates in Sanya and northern China on asthma and allergic diseases, and explores the impact of climate on asthma, chronic obstructive pulmonary disease (COPD) and allergic diseases.

2. What is the main research content, research protocol of this research?

We regularly collect factors such as diet, living and living environment, and family history that may affect the occurrence of asthma, COPD and allergic diseases for screening suspected allergic diseases.

3. What do you need to do if you participate in this research?

Please cooperate with filling out the questionnaire and the physical examination including collection of venous blood for blood routine examination and other routine examinations.

4. What are the possible benefits for you to participate in this study?

Middle-aged and elderly people can take free physical examinations, including blood routine, blood sugar, blood lipids, liver and kidney function, lung function, etc., and to track the development and changes of diseases such as asthma, allergic rhinitis, chronic obstructive pulmonary disease, etc. The subjects have no obvious potential discomfort or risk.

5. What adverse reactions, risks and discomforts may I face if I participate in this study?

There may be temporary local pain while sampling and filling out the questionnaire may take a small amount of your time. There will be no other obvious adverse reactions or other risks.

6. If you choose to participate in the study, can you ask to quit midway?

Participation in the study is entirely at your discretion, and you may refuse to participate in the study or request to withdraw from the study at any time during the study.

7. If you participate in the study, will your personal information be disclosed?

During the research process, all your relevant information (including personal information, etc.) will be completely stored in the institution where the doctor (researcher) is located. No one other than your attending physician, investigator or ethics committee is permitted to access the above information. Any public reporting of the results of this study will not disclose your personal identity. We will make every effort to protect the privacy of your personal medical information to the extent permitted by law.

8. If you participate in this research, who can you contact if you have any questions during the research process?

You can ask any questions about this research at any time to get answers by contacting the project contact person responsible for this research. If there is any important new information during the research process that may affect your willingness to continue participating in the research, the above-mentioned personnel will also communicate with you in a timely manner.

10. Others

It is up to you (and your family) to decide whether to take part in this study. Please ask your doctor as many questions as possible before making your decision to participate in the study.

Thank you for reading the above material. Please sign below if you decide to participate in this study.

Project Contact: Ren Han

Contact: 15595980629

Statement of Consent

I have read the above description of the study and have had the opportunity to discuss and ask questions about the study with the project staff. All my questions have been answered satisfactorily.

I am aware of the possible risks and benefits of participating in this study. I understand that participation in research is voluntary, I confirm that I have had sufficient time to consider this, and I understand that I can ask the person responsible for the research for more information at any time, and I can ask to withdraw from the research at any time without discrimination or retaliation, the rights will not be affected.

I decided to agree to participate in this research and promise to actively cooperate with the project team to complete the project-related physical examination and fill in the questionnaire. I will get a signed and dated copy of the informed consent form.

Name:____________________ Date of Signature:______Year____Month____Day

调查人员：①HR( )②QH( )③HDG( )④DYC( )⑤YCH( )⑥KQZ( ) ⑦自填( )⑤其他：

中老年健康调查问卷

亲爱的中老年朋友，登记编号：_______姓名：_________，性别：① 男 ② 女,出生日期:____年___月___日，

1.原居住地：①黑龙江 ②吉林省 ③辽宁省 ④内蒙古 ⑤新疆 ⑥甘肃省 ⑦陕西省 ⑧北京市 ⑨河北省 ⑩天津 （11）山西省 （12）其他：_______省；（13）三亚本地长期居住 5 年以上

2.您目前是否患有下列疾病 （根据所患疾病史选择现有疾病状况进行询问）

①哮喘（ ）；②慢性阻塞性肺病（慢阻肺）（ ）；③过敏性鼻炎/咽炎（ ）④湿疹/荨麻疹/皮炎（ ） ⑤食物/药物过敏 ⑥支气管炎/支气管扩张 （ ） ⑦肺气肿 （ ）⑧高血压（ ）⑨糖尿病（ ） ⑩其他___________

3.体检清单，请在完成的项目之后打勾或填写结果。

A.身高：______cm，B.体重：_____kg；C.血压：收缩压：______mmHg，舒张压：_______mmHg；D.肺功能：( )

第一部分 基本情况

4.现居住地:三亚吉阳区①左岸凤凰水城 ②翠屏凤凰水城 ③万科湖畔 ④一山湖 ⑤悠岚湖 ⑥子悦台⑦其他__。

5.海南居住 3 个月以上总共__次，居住三亚__次，常规每年__月来，次年___月离开，一年四季常住（ ）

6.现在的住房是买的还是租的：①购买的：自己或子女购买（ ），其他亲戚购买（ ），②租房（ ）；

7.文化程度：①文盲或半文盲 ②小学 ③初中 ④中专、技校 ⑤高中 ⑥大专 ⑦本科 ⑧研究生（硕士或博士）

8.您退休前的职业：①机关行政管理人员；②企业管理人员；③私营企业主；④专业技术人员 ；⑤普通职员；⑥个体户；⑦商业服务人员；⑧工人（生产、运输设备操作人员及有关人员 ）

⑨农民、林、渔、牧、水利生产人员；⑩无业或失业

9.您是否经常查看微信群通知或消息：①是 ②否；您希望下次调查通知方式：①微信群通知（） ②电话通知（）

您的联系电话(非常重要)：________________________；您的家人电话(非常重要)：________________________；

第二部分 既往病史及过敏性疾病症状

一、 既往病史和家族史

10.您 **18 岁前**是否患有呼吸道疾病或慢性咳嗽？（ ），如果有，①12 岁之前（ ），② 12 岁之后（ ）

11.**家庭成员中**是否有下列呼吸道疾病史：①哮喘（ ）；②慢性阻塞性肺病（慢阻肺）（ ）； ③过敏性鼻炎（ ）④湿疹/皮炎（ ）⑤食物/药物过敏（ ） ⑥支气管炎/支气管扩张 （ ） ⑦肺气肿 （ ）⑧其他呼吸道疾病（ ）。

二、 现有疾病状况（请只填写病史中对应的疾病）

（一）关于哮喘

12. 在北方居住您是否有以下症状：①胸部喘息或口哨音；②因胸闷，呼吸急促，或因咳嗽发作而夜间醒来

13.与北方原居住地相比，您到三亚之后哮喘病情有何变化：

①明显缓解 ②有所缓解 ③无明显变化 ④加重 ⑤明显加重

若选择明显缓解或有所缓解，是症状减轻：（ ），发作次数减少：（ ），服药减少或停药：（ ）

（二）关于慢性阻塞性肺病（COPD）

14.您是否出现下列呼吸症状：① 咳嗽（ ）②咳痰（ ） ③喘息（ ） ④呼吸困难（ ）⑤咯血（ ）⑥其他（ ）

15.与北方原居住地相比，您到三亚之后慢性阻塞性肺病病情有何变化：

①明显缓解 ②有所缓解 ③无明显变化 ④加重 ⑤明显加重

若选择明显缓解或有所缓解，是症状减轻：( )，发作次数减少：( )，服药减少或停药：( )

（三）关于过敏性鼻炎/咽炎

16. 在北方原居住地，在没有感冒或流感的情况下，您是否有过①打喷哽（ ）②流涕（ ）③鼻塞（ ）④鼻痒（ ）⑤眼痒（ ） ⑥眼干（ ）⑦咳嗽（ ）⑧咳痰（ ）⑨咽干（ ）⑩咽痛（ ）

17. 上述症状：①是否干扰睡眠？（ ）②是否限制日常活动（运动、休闲）？（ ）③是否让您觉得烦恼？（ ）

18. 每周平均____天有鼻部或咽部症状或最严重时症状如何？

19. 在北方原居住地，鼻部或咽部症状能持续时间：① 连续<4 周（ ） ②连续>4 周（ ）

20. 总体上看您有鼻腔或咽部症状___年？

21. 您是否在每年相同的季节有鼻部症状吗？（ ）如果是，每年__月份症状加重？

22. 您知道是什么原因引起您的鼻部或咽部症状吗？ （可以多选）

①接触植物或花粉 ②接触冷空气 ③接触宠物 ④接触油漆、粉尘、烟雾 ⑤其他

23. 在北方原居住地，您是否因为过敏性鼻炎或咽炎去过医院或诊所？（ ）

24. 您因为过敏性鼻炎或咽炎接受过：①手术治疗 ②药物治疗 ③ 均无

治疗后感觉如何？ ① 有效 ② 无效 ③ 效果不明显

25. 目前是否还在用药？① 是 ② 否

26. 您的鼻部或咽部问题伴随这些疾病吗？ ① 哮喘 ② 湿疹 ③ 鼻息肉/鼻窦炎 ④ 过敏性咳嗽

27. 您的家人有过敏性疾病困扰吗？ ①有 ② 没有

28. 与原居住地相比，您到三亚之后过敏性鼻炎或咽炎病情有何变化：

①明显缓解 ②有所缓解 ③无明显变化 ④加重 ⑤明显加重

若选择明显缓解或有所缓解，是症状减轻：( )，发作次数减少：( )，服药减少或停药：( )

29. 您是否经常游泳：① 是 ② 否；游泳之后鼻炎是否加重：① 是 ② 否

（四）关于湿疹/荨麻疹/皮炎

30. 您是否有过或被诊断为湿疹? ①是 ②否

31. 您是否有过或被诊断为荨麻疹? ①是 ②否

32. 除湿疹和荨麻疹外，您是否被诊断为其他类型的皮炎? ①是 ②否

33. 您既往是否有过反复的皮肤瘙痒或关节屈侧皮疹 ①是 ②否；是否有其他部位皮疹：①是 ②否

a. 若回答为“是”，此现象在您___岁时首次出现

b. 若回答为“是”，此现象是否与食物、环境、天气等改变有关 ①是 ②否

c. 在最近 12 个月，您是否有过反复皮肤瘙痒或关节屈侧皮疹 ①是 ②否

34. 与原居住地相比，您到三亚之后湿疹、荨麻疹或特应性皮炎病情有何变化：

①明显缓解 ②有所缓解 ③无明显变化 ④加重 ⑤明显加重

若选择明显缓解或有所缓解，是症状减轻：( )，发作次数减少：( )，服药减少或停药：( )

（五）关于食物/药物过敏

35.在北方您是否曾被医生诊断为食物过敏 ① 是 ② 否

36.您是否做过过敏原检测？ ① 是 ② 否 37.检测结果显示或生活中是否发生过下列食物过敏？

①牛奶或奶制品 ②鸡蛋 ③鱼虾蟹 ④坚果 ⑤小麦 ⑥牛肉 ⑦豆类或豆制品 ⑧水果 ⑨蔬菜 ⑩其他

38. 您有无接受过青霉素等抗生素类药物的治疗?(可多选)

（1）① 无 ② 有，（2）①1 岁以内 ②1-2 岁 ③ 3 岁以后；（3）这样的治疗有__次?

39. 您是否曾被医生诊断为药物过敏 ① 是 ② 否 40. 您有无药物过敏的表现 ① 有 ② 无

如有，为以下哪些（可多选）：a.青霉素（ ）b.头孢菌素类（ ）c.磺胺类（ ）d.其它（ ） e.不确定（ ）

（六）关于支气管炎/支气管扩张/肺气肿

41. 在北方您是否有连续 2 年中，每年 3 个月以上的咳嗽或咳痰？①是（ ） ②否（ ）

42. 您是否在医院或医生诊断过支气管扩张或肺气肿？①是（ ） ②否（ ）

43. 与原居住地相比，您到三亚之后支气管炎/支气管扩张/肺气肿病情有何变化：

①明显缓解 ②有所缓解 ③无明显变化 ④加重 ⑤明显加重

若选择明显缓解或有所缓解，是症状减轻：（ ），发作次数减少：（ ），服药减少或停药：（ ）

（七）关于高血压

44. 与原居住地相比，您到三亚之后高血压病情有何变化：

①血压恢复正常 ②血压有所下降但仍然偏高 ③无明显变化 ④血压上升

是否服用降压药：①血压下降后不再服药（ ） ②血压下降药量减少（ ）③药量不变化（ ）

第三部分 行为习惯和环境暴露

一、行为习惯

45. 您每周吃海鲜的频率：①不吃 ②小于 1 次/月 ③小于 1 次/周 ④1-3 次/周 ⑤4-6 次/周 ⑥1 次/天

46. 您是否抽烟？ ①以往抽过目前戒烟，以往抽过大约___年？每天抽__支 ②目前抽烟，已经抽烟____年？每天抽__支；③不抽烟也无被动吸烟；④不抽烟，但是家庭或工作场所有人经常抽烟（被动吸烟)。

47. 您每天运动吗？ ①散步____万______千步/天或___小时； ②跳舞（ ） ③打拳（ ） ④其他（ ）

48. 您的睡眠状况，每天晚上___点睡觉，早上___点起床，午睡____小时；

二、原居住地工作和居住环境情况

49.您以往工作中是否有长时间职业粉尘或气体暴露 ①有， ②没有

50.原居住地住房周围 50 米的车流状况 ①靠近主要交通干道 ②距离交通干道较远小区 ③偏远小区

51.原居住地房屋类型：①楼房，您家住____楼. ②自家盖楼房 ③平房 ④别墅 ⑤其他，

52. 您家中饮用水的主要来源是？ ①自来水 ②山泉水 ③桶装纯净水 ④净水器处理水 ⑤井水 ⑥地下水

53. 您原居住地家室内是否养花草？ ①是 ②否；50. 您家中是否有毛绒玩具 ①是 ②否

54. 您家厨房或加热采用什么燃料?①集中供暖等；②空调或地暖； ③现代燃料，如液化气、煤气、天然气；④生物燃料，如木材、农作物、动物粪便或木炭等；

55. 您家厨房的通风情况 ①很差 ②一般 ③良好 ④很好

56.近一年来，家中有霉味或刺激性气味吗？①没有 ②几乎天天有 ③偶尔有 ④仅下雨天有 ⑤仅梅雨季节有

57.目前您家中是否有宠物？ ①没有 ②有；您是否由于家庭成员过敏性疾病而处理掉宠物？ ①是 ②否

58.原居住地近一年，您曾在家中是否看到过老鼠或蟑螂？ ①没有 ②很少 ③较少 ④很多

59. 您一般多久对房间进行清洁(包括吸尘、扫地、拖地等)？

①每天 ②一周 4-6 次 ③一周 1-3 次 ④每月 1-3 次 ⑤少于每月一次

60.您家的窗帘隔多少时间洗一次？ ①从未清洁 ②每月 ③每季 ④半年 ⑤一年及以上

知情同意书

尊敬的居民朋友：

我们邀请您参加一项名为“三亚市气候环境因素对哮喘等呼吸系统疾病影响的研究”项目。 在您决定是否参加该项研究之前，请仔细阅读以下内容，它可以帮助您了解该项研究的研究目的、研究内容、研究方案和和研究期限，以及如果参加该项研究可能给您带来的受益、风险或不适。您可以与您的亲属、朋友一起讨论，或者请项目组工作人员给予更详细的说明，以帮助您做出是否加入本项研究的决定。

1、为什么要进行该项研究？

哮喘和过敏性疾病是威胁人类健康的重要疾病。本项目分析三亚市气候环境因素对中老年哮喘和过敏性疾病的影响；对比三亚和北方不同气候环境对哮喘、过敏性疾病的影响差异，探讨气候环境对哮喘、慢性阻塞性肺病（COPD）和过敏性疾病的影响。

2. 该项研究的主要内容和研究方案是什么？

我们会定期收集可能影响哮喘、慢阻肺和过敏性疾病发生的饮食、生活及居住环境、家族史等因素，用于筛选过敏性疾病疑似人员。

3、如果您参加该项研究，需配合做些什么事情？

请您配合填写调查问卷并配合采集静脉血检测血常规等体检相关常规检查。

4、如果参加该研究，您可能获得哪些受益？

中老年人可以**免费接受体检相关的检查，包括血常规、血糖、血脂、肝肾功能、肺功能等，跟踪哮喘、过敏性鼻炎、慢性阻塞性肺病等疾病发展变化**，受试者无明显的潜在不适或风险。

5. 如果参加该项研究，可能会面临什么不良反应、风险和不适？

采样时可能会带来局部暂时疼痛，填写调查问卷可能需要占用您少量时间。不会产生其它明显不良反应或面临其它风险。

6. 如果您选择参加该项研究，能否要求中途退出？

是否参加研究完全取决于您的意愿，您可以拒绝参加此项研究，或在研究过程中的任何时间要求退出本研究。

7、如果您参加该项研究，个人信息是否会被泄露？

在研究过程中您所有的相关资料（包括个人信息等）均将完整地保存在医生（研究者）所在机构。除了您的主治医生、研究者或伦理委员会被允许查阅上述资料外，任何其他人员均不能查阅。任何有关本项研究结果的公开报告将不会披露您的个人身份。我们将在法律允许的范围内，尽一切努力保护您个人医疗资料的隐私。

8、如果您参加该项研究，在研究过程中如有问题可与谁联系？

您可以在任何时间提出有关本项研究的任何问题，并得到相应的解答，具体可与负责本项研究的项目联系人联系。如果在研究过程中有任何重要的新信息，可能影响您继续参加研究的意愿时，上述人员也会及时与您沟通。

10、其他

是否参加本项研究由您自己（和您的家人）决定。在您做出参加研究的决定前，请尽可能向你的医生询问有关问题。

感谢您阅读以上材料。如果您决定参加本项研究，请在下方签字。

项目联系人：任 寒

联系方式：15595980629

同意声明

我已经阅读了上述有关本研究的介绍，而且有机会就此项研究与项目人员讨论并提出问题。我提出的所有问题都得到了满意的答复。

我知道参加本研究可能产生的风险和受益。我知晓参加研究是自愿的，我确认已有充足时间对此进行考虑，而且明白可以随时向负责该项研究的人员咨询更多的信息，也可以随时要求退出本研究而不会受到歧视或报复，权益不会受到影响。

我决定同意参加本项研究，并保证积极配合项目组完成项目相关的体检并填写问卷。我将获得一份经过签名并注明日期的知情同意书副本。

姓名：____________________ **签署日期:** _______年_____月_____日
